# Cryopreservation and replantation of amputated rat hind limbs

**DOI:** 10.1186/2047-783X-19-28

**Published:** 2014-05-23

**Authors:** Zengtao Wang, Bo He, Yongzhuang Duan, Yun Shen, Lei Zhu, Xiaolei Zhu, Zhaowei Zhu

**Affiliations:** 1 Department of Orthopaedic and Microsurgery, The First Affiliated Hospital of Sun Yat-sen University, 58 Zhongshan Road 2, Guangzhou 510080, China; 2 Department of Hand and Foot Surgery, Provincial Hospital Affiliated to Shandong University, Jinan 250021, China; 3 Department of Orthopaedics, The First Affiliated Hospital of Zhengzhou University, Zhengzhou 450052, China; 4 The Science and Technology Research Institute of the National Population and Family Planning Commission, 12, Dahuishi Road, Haidian District, Beijing, China; 5 Department of Hand and Foot Surgery, Qilu Hospital of Shandong University, Jinan 250012, China

**Keywords:** Amputation, Cryopreservation, Injury, Rat model, Replantation

## Abstract

**Background:**

In spite of the relatively high success rate of limb replantation, many patients cannot undergo replantation surgery because the preservation time of an amputated limb is only about six hours. In addition, although allotransplantation of composite tissues is being performed more commonly with increasingly greater success rates, the shortage of donors limits the number of patients that can be treated. So the purpose of this study is to examine the feasibility of cryopreservation and replantation of limbs in a rat model.

**Methods:**

Twelve five-month-old Sprague-Dawley rats were divided evenly into group A (above-knee amputation) and group B (Syme’s amputation). One hind limb was amputated from each rat. The limbs were irrigated with cryoprotectant, cooled in a controlled manner to -140°C, and placed in liquid nitrogen. Thawing and replantation were performed 14 days later.

**Results:**

In group A, the limbs became swollen after restoration of blood flow resulting in blood vessel compression and all replantations failed. In group B, restoration of blood flow was noted in all limbs after replantation. In one case, the rat chewed the replanted limb and replantation failed. The other five rats were followed for three months with no abnormalities noted in the replanted limbs.

**Conclusions:**

Limbs with a minimal amount of muscle tissue can be successfully cryopreserved and replanted.

## Background

Since 1949, when British biologist Christopher Polge found that glycerol-treated sperm could survive after being frozen at low temperature [1], cryopreservation has been extensively developed and widely applied in clinical medicine. Cryopreservation is distinguished from freezing in that cryoprotective agents are added to preserve the biological integrity of the tissues [2,3]. Freezing of tissue, such as bone, is done without the use of cryoprotective agents and usually the temperature is not less than -80°C. On the other hand, the intent of cryopreservation is to place the tissues in a state of suspended animation in which biological processes are suspended, and the final temperature is typically that of liquid nitrogen, -196°C [2-4]. Cryopreservation is routinely used for the preservation of homogenous cell populations and single-cell layer tissues [3]. While still in experimental stages, advances are being made in the cryopreservation of whole organs. In 2002, Wang *et al*. [5] preserved rat ovary using cryopreservation techniques, and successfully performed *in situ* replantation after thawing. Yin *et al*. [6] have reported the successful cryopreservation and replantation of rat testes and ovaries, and Tanaka *et al*. [7] reported the successful transplantation of cryopreserved tracheal allograft in a rabbit model. Other authors have reported the successful cryopreservation of hepatocytes [8,9] and whole murine and porcine livers [10].

While it is possible to cryopreserve various types of tissues, and subsequently thaw the preserved specimens and achieve functionality, the optimal parameters for cryopreservation (for example, vitrification fluid, cooling rate) vary for different tissue types [4]. Composite tissues, such as a limb, are composed of a number of tissue types including skin, muscle, vessels, nerves, and bone. Thus, cryopreservation of composite tissues is extremely challenging and remains in its infancy, though advances are continually being made [11,12].

Replantation and allotransplantation of composite tissue such as reattachment of a severed limb or cadaveric hand transplantation has seen marked advances in the past decade [13-15]. However, the number of donors is limited, composite tissue must be transplanted shortly after removal, and in many cases of traumatic amputation a patient is not stable enough to undergo replantation. Thus, cryopreservation of composite tissue, though technically challenging, holds the promise of improving the quality of life for a large number of people. In addition, evidence suggests that cryopreserved tissues decreased immunogenicity, and thus allotransplants are less likely to initiate an immune response in the recipient [7].

We successfully preserved amputated limbs of rats in liquid nitrogen using cryopreservation techniques, providing a practical basis for long-term preservation of amputated limbs at low temperature [16]. The purpose of the study was to examine the feasibility of the replantation of cryopreserved limbs using a rat model.

## Methods

### Determination of cryoprotectant concentration

Human SV40 transfected osteoblasts were cryopreserved with programed cooling with different concentrations of dimethyl sulfoxide (DMSO) for observation of the changes during the process. For this experiment the reagents included: human SV40 transfected osteoblasts; DMEM; F12 medium; G418; FBS; annexin V/PI fluorescent double labeling reagent kit.

The culture medium was composed of: 1) a 1:1 mixture of DMEM medium containing 2.5 mM of L-glutamine, 15 mM of HEPES, 0.5 mM of pyruvic acid and 1.2 mg/L of sodium carbonate, and F12 medium accounting for 90 % of the total volume; 2) 0.3 mg/ml of G418; and 3) FBS (10%). The cell line was cultured in the aforementioned culture medium at 37°C with saturated humidity in a 5% CO_2_ incubator. After culture, the cells were divided into three groups and placed in various concentrations of DMSO: 0% (B0), 10% (B1), and 50% (B2) DMSO concentration. Ten minutes after placing the cells in the DMSO, cryopreservation of the cell/DMSO mixture was performed using the programed cooling method subsequently described for limb cryopreservation.

After cryopreservation, the cryopreservation vial was removed from the liquid nitrogen and place in a 40°C water bath for de-freezing. After thawing, the percentage of apoptosis, necrosis, and cell viability was determined using annexin V/PI fluorescent double-labeling and double-parameter flow cytometry analysis.

### Experimental animals and grouping

All animals in this study were cared for, and experiments were performed according to, established guidelines for the use and care of laboratory animals. This study was approved by the Institutional Review Board and the Animal Care and Use Committee of our institution, The First Affiliated Hospital of Sun Yat-sen University, The Provincial Hospital Affiliated to Shandong University.

Twelve healthy adult male Sprague-Dawley rats approximately five months of age and weighting 200 to 250 g were selected. Animals were obtained from Experimental Animal Center of Shandong University, and housed in a 12 hour light/dark cycle and fed standard rat chow and water *ad libitum*. The 12 rats were divided evenly into group A (above-knee amputation) and group B (Syme’s amputation).

### Limb harvesting

**Figure 1:**
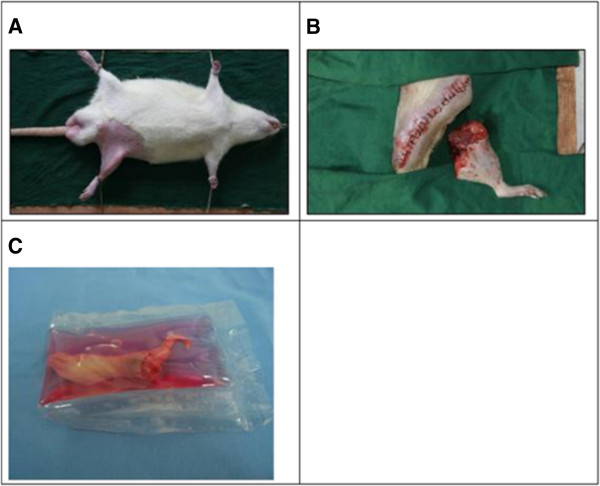
**Limb harvesting. (A)** Preoperative preparation. **(B)** Amputated limb. **(C)** Amputated limb in cryopreservative before cooling.

Anesthesia consisting of ketamine (30 mg/kg) was injected into the abdominal cavity of the rat, and the rat was fixed on the operating table. Skin preparation was carried out on the bilateral thighs. Only one hind limb was amputated from each rat (Figure 1).

In group A, a circular skin incision was made at the upper one third of the thigh medially, and the subcutaneous fascia was dissected. The femoral artery and vein were dissected for a length of about 1 cm. Microvascular clips were placed on the proximal end of the artery to block blood flow. The femoral artery and vein were ligated, and the muscles, femoral nerve, and sciatic nerve were then cut to reach the femur. The femur was cut with a saw after periosteal dissection, thus establishing an above-knee amputation model. In group B, a circular skin incision was made around the ankle medially, and the subcutaneous fascia was dissected. The posterior tibial artery and vein were dissected for a length of about 1 cm. Microvascular clips were placed on the proximal end of the artery to block blood flow. The posterior tibial artery and vein were ligated, and the muscles and nerves were cut to reach the ankle joint. The ankle joint was disarticulated after periosteal dissection, thus establishing a Syme’s amputation model. After amputation, part of femur or tibia was shortened. The stump was covered with peripheral soft tissue, and the skin was sutured directly.

### Cryopreservation

**Figure 2:**
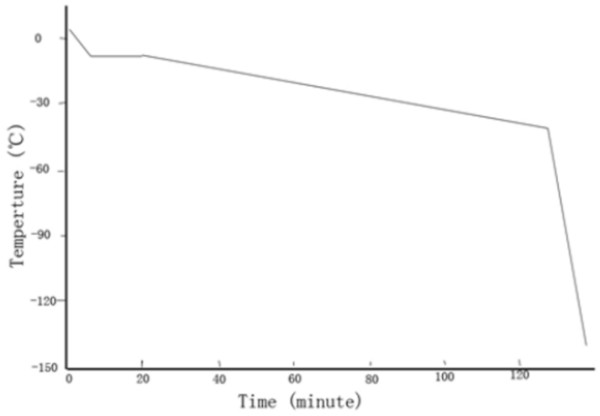
Cooling curve.

All limbs were cryopreserved immediately after amputation. Before cryopreservation the amputated limbs were irrigated with normal saline, followed by perfusion with cryoprotectant (10% FBS, 10% DMSO, and 5% sucrose RPMI1640 medium) at 4°C at a rate of 50 ml/hour for 15 minutes. The cryoprotectant was perfused until residual blood in the limb was washed completely out, and the cryoprotectant was drained from the vein. After 20 minutes of immersion and balancing, the limbs were sealed in individual plastic bags for preservation. The sealed bag was then placed in a programed freezing device for cooling (Figure 2), and subsequently placed in liquid nitrogen for preservation.

### Thawing process

A total of 200 ml eluent (RPMI1640 medium) was placed in a sterile beaker in a 40°C water bath. After the eluent reached a temperature of 40°C, the frozen limb was taken out of the liquid nitrogen and put into the eluent for a period of eight minutes. The rewarmed limb was perfused with RPMI1640 medium at 50 ml/hour for 15 minutes. Perfusion buffer was drained from the vein.

### Replantation

In both groups, replantation was carried out 15 days after the limb was cooled and preserved. Rats were anesthetized by an intraperitoneal injection of 3% pentobarbital sodium (30 mg/kg). The original incision of the stump was approached, and the femur (group A) or tibia (group B) respectively, was exposed. After locating the femoral artery (group A) or anterior/posterior tibial artery (group B), femoral artery-femoral artery or anterior/posterior tibial artery-anterior/posterior tibial artery arterial anastomosis was performed. In both groups, end-to-end anastomosis was performed for the arteries and veins. In group A, vessels were sutured after fixing the bone with a Kirschner wire. In group B, the limb was fixed by transversing a 1 ml syringe through the tibia. After suturing anterior and posterior tibial tendon and vessels, the skin was loosely sutured.

All rats received papaverine (15 mg, q4h) and penicillin (40,000 U in 5 ml normal saline, twice daily) by intramuscular injection for the first 5 days after surgery. Rats were maintained under a heat lamp at 26°C for 10 days. Limb survival 14 days after surgery was considered successful replantation.

### Electron microscopy of replanted muscle tissue

Electron microscopy was performed of the gastrocnemius of the hind limb from a severed thigh from group A (above-knee amputation), which was fixed in 4% glutaraldehyde. The sample was dehydrated, embedded, and sliced according to routine transmission electron microscope sample preparation methods. The skeletal muscle ultrastucture was observed under transmission electron microscopy.

### Statistical analysis

Data were presented a number (percentage) or median ± standard deviation. Differences in limb survival were compared with Fischer’s exact test. The comparison of the averages of two samples for the analysis of cell viability was analyzed with the paired *t*-test. Analysis was performed with SPSS statistics software (SPSS Inc., Chicago, IL, USA), and a value of *P* < 0.05 was set as statistical significance.

## Results

### Cryoprotectant concentration

**Figure 3:**
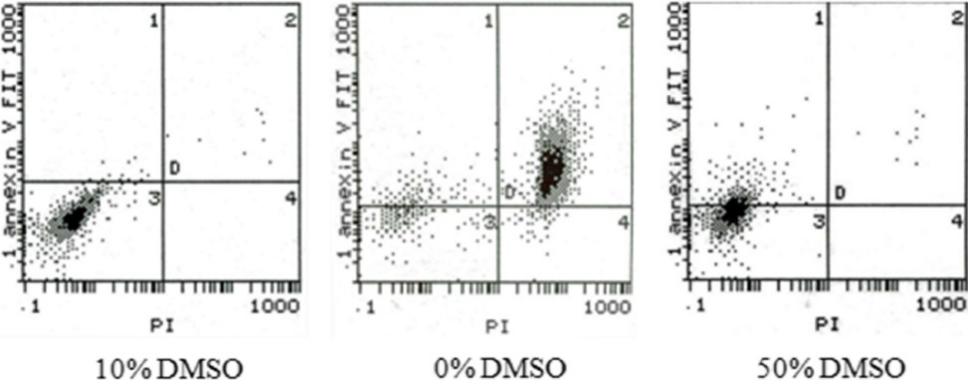
**Scatter plot of double-parameter flow cytometry.** Human SV40 transfected osteoblasts; dots at lower left quadrant (H3) were viable cells (FITC-/PI-); dots at upper right quadrant (H2) were non-viable cells, which were necrotic (FITC+/PI+); dots at upper left quadrant (H3) were apoptotic cells (FITC+/PI-).

Results are summarized in Table 1 and Figure 3. Cell necrosis and debris increased after freezing without a cryoprotectant (group B0), as intracellular ice formation damaged the cytomembrane. Cell viability was greatest in group B1, indicating that 10% DMSO provided a good protective effect. In group B2 (50% DMSO) the cell viability was decreased with obvious apoptosis, but cell necrosis and debris was reduced, which suggested that a high concentration of DMSO can induce cell defects finally resulting in apoptosis. Thus, a concentration of 10% DMSO was used for limb cryopreservation.

**Table 1:** Comparison of apoptosis, necrosis and cell debris, and cell viability of cells preserved with different concentrations of dimethyl sulfoxide (DMSO)

**Groups**	**Apoptosis (%)**		**Necrosis and cell debris (%)**		**Cell viability (%)**	
	**Before**	**After**	**Before**	**After**	**Before**	**After**
	**Cryopreservation**		**Cryopreservation**		**Cryopreservation**	
B0	7.7 ± 0.7	8.2 ± 5.9	2.8 ± 0.4	88.0 ± 6.2^a^	89.5 ± 3.1	3.8 ± 1.6^a^
B1	7.4 ± 0.8	7.4 ± 1.5	3.2 ± 0.9	3.8 ± 0.9	89.4 ± 3.7	88.8 ± 0.9
B2	8.1 ± 0.7	37.5 ± 2.1^a^	3.1 ± 0.7	4.3 ± 0.9	88.2 ± 3.5	58.3 ± 2.6^a^

Data are presented as mean ± standard deviation.

B0, 0% DMSO; B1, 10% DMSO; B2, 50% DMSO.

Sample size = 7.

^a^*P* < 0.05 compared to before cryopreservation.

### Limb cryopreservation

**Figure 4:**
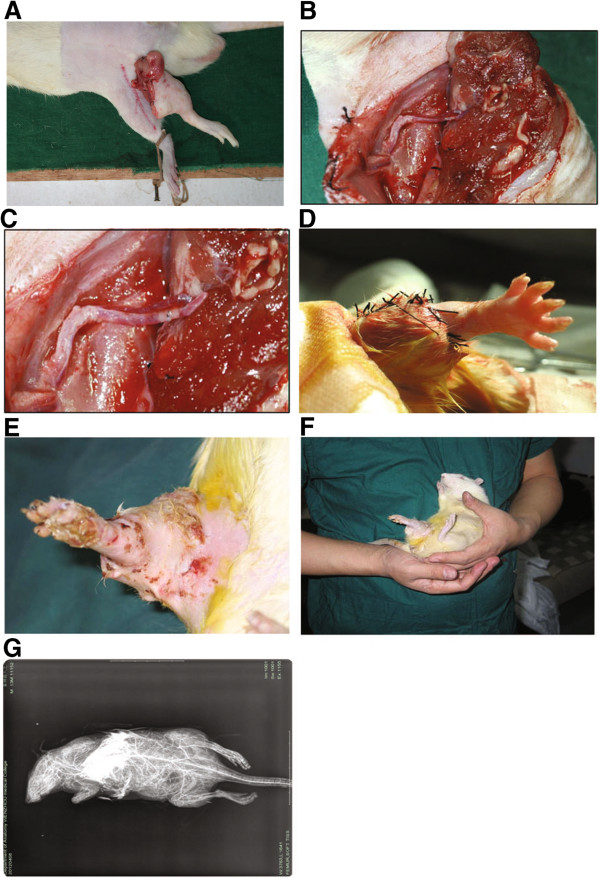
**Intra- and postoperative images. (A-C)** Above-knee amputation group; **(A)** Limb post freezing and rewarming is swollen compared to the healthy limb. **(B)** Immediately after anastomosis and **(C)** attempted restoration of blood flow marked muscle swelling was noted. **(D-G)** Syme’s amputation group; **(D)** Immediately after replantation the limb is pink indicating adequate blood flow. **(E)** Ten days after replantation no signs of necrosis were noted. **(F)** At two months after replantation the limb is viable. **(G)** Angiography at two months after surgery showed blood flow in the transplanted limb, though a reduced number of blood vessels.

Replantation failed in all six rats in group A (above-knee amputation), whereas replantation was successful in five out of six rats (83%) in group B (Syme’s amputation). The case that failed in group B was because the rat bit and chewed the replanted limb. The difference in limb survival (0 versus 5) was significant (*P* < 0.05, Fisher’s exact test).In group A, after restoration of blood flow the limbs became swollen resulting in compression of the artery and vein until embolism occurred. Ultimately, blood flow ceased after six to eight hours and replantation failed due to repeated embolization in the artery as a result of compression (Figure 4A-C).In group B, restoration of blood flow was noted in all limbs after replantation. In one case in which the rat chewed the replanted limb, replantation failed. In the other five cases, the rats were able to crawl without difficulty and the rats were followed for three months with no abnormalities noted in the replanted limbs at which time they died of natural causes (Figure 4D-F). Angiography of an animal in group B at two months after surgery shows blood flow in the transplanted limb, though a reduced number of blood vessels (Figure 4G).

### Electron microscopy of replanted muscle tissue

**Figure 5:**
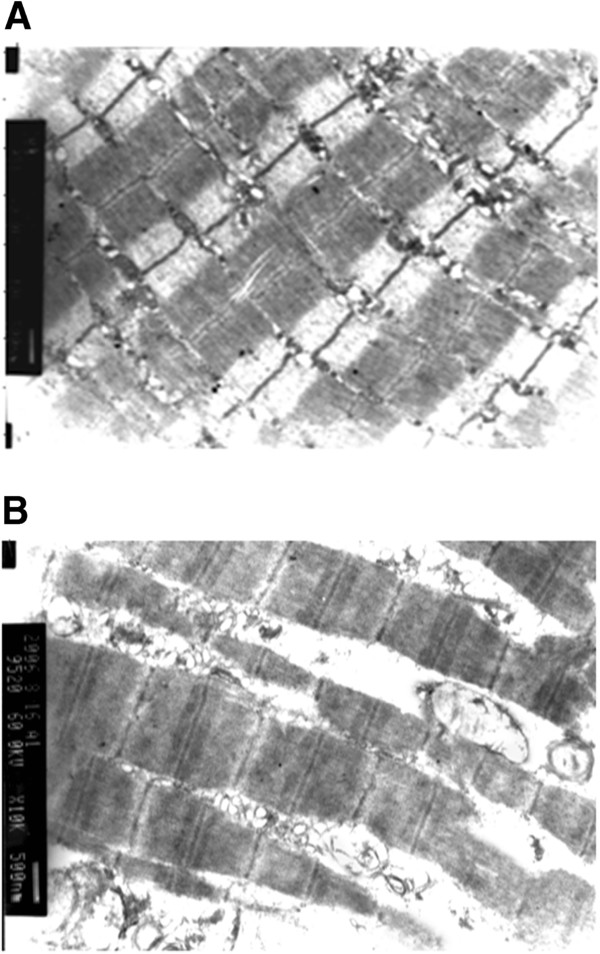
**Electron microscopy. (A)** Normal skeletal muscle obtained after severing the rat limb but before freezing. Regularly arranged myofibrils, clear z-lines, dense mitochondrial cristae, and intact mitochondrial membranes without swelling were seen. **(B)** Skeletal muscle of a failed replantation after cryopreservation. Images were obtained four hours after reperfusion. Some myofibrils were ruptured with unclear z-lines, the mitochondria exhibited maximal swelling, crests were disarrayed, and parts of the myofibrils showed vacuolation.

Electron microscopy images of normal skeletal muscle and skeletal muscle that failed replantation are shown in Figure 5. In normal muscle tissue, regularly arranged myofibrils, clear z-lines, dense mitochondrial cristae, and intact mitochondrial membranes without swelling were seen. In muscle tissue that failed replantation, some myofibrils were ruptured with unclear z-lines, the mitochondria exhibited maximal swelling, crests were disarrayed, and parts of the myofibrils showed vacuolation.

## Discussion

In spite of the relatively high success rate of limb replantation, many patients cannot undergo replantation surgery because the preservation time of an amputated limb is only about six hours. For example, many patients with associated injuries to vital organs cannot undergo replantation because their physical condition is not suitable for replantation surgery. In addition, although allotransplantation of composite tissues is being performed more commonly with increasingly greater success rates, the shortage of donors limits the number of patients that can be treated [14].

If the metabolic processes of tissues can be slowed, the occurrence of degeneration and necrosis can be delayed, thus allowing time for the treatment of other medical conditions; subsequent replantation of the amputated limb or allotransplantation from an appropriate donor can then occur. Nakagawa *et al*. [17] found that the maximum ischemia time for replantation of a limb containing muscle tissue was prolong to 8 hours at -1°C, but the maximum ischemia time at 4°C could not be prolonged to 8 hours. Kour *et al*. [18] reported two successful cases of major replantation of the upper extremity using a technique of complete vascular washout with 4°C University of Wisconsin cold storage solution in which the ischemia times of the amputated limbs were 7 hours and 11.25 hours. Kato *et al*. [19] reported that rat hearts preserved in a variable magnetic field at -3°C for 24 hours exhibited satisfactory hemodynamic and metabolic performance. Preservation as described above only reduces the metabolic needs of tissues, and is thus not suitable for long-term storage.

The preservation time of dynamic tissues and organs is related to the preservation temperature, and in general the lower the temperature the longer the preservation time [20,21]. Arrhenius indicated that the chemical reaction rate constants changed with the temperature, and their relationship was written as:

k=Aexp-Ea/RT

where k is the reaction rate constant, R is the molar gas constant, Ea is the apparent activation energy, and A is the pre-exponential factor (also called frequency factor). Based on this formula, tissues could be stored alive for 2 hours at 4°C, several days at -40°C, several months at -80°C, and theoretically for several centuries at -196°C. The deep low temperature mentioned in our study usually refers to a temperature below -80°C.

While the challenges of cryopreservation of composite tissues are daunting, reports have begun to show the feasibility of the process. We preserved an amputated finger in liquid nitrogen for 81 days using a programed cooling method and successfully performed replantation [22], thus proving that biological materials can survive at low temperature. Hirasé *et al*. [23] introduced a long-term storage method for vessels and nerves that extended the possibilities for clinical use of cryopreserved vessels and nerves using replantation in a rat model. In this study we examined the feasibility of limb cryopreservation and replantation in a rat model. In the group that received Syme’s amputations, five of six cryopreserved and replanted limbs survived to the last follow-up point of three months. However, replantation of all six cryopreserved limbs that received an above-knee amputation failed in the immediate postoperative period as a result of absent blood flow due to arterial compression from swollen muscle. Bakhach [11] reported the successful xenotransplantation of cryopreserved human digits that did not meet the criteria for microsurgical revascularization in a rabbit model. Rinker *et al*. [12] harvested epigastric flaps from Lewis rats which were then cryopreserved with a DMSO/trehalose cryoprotective agent, frozen to -140°C by controlled cooling, and stored for 14 days. The flaps were then thawed and replanted, and nine of ten flaps were viable at sixty days after replantation.

Currently, the common cyroprotectant used for cells and tissue is DMSO. Although DMSO protects cells from cryo-damage, it also is toxic to cells. The results of this study showed that without cyroprotectant (B0), intracellular ice formation might penetrate the cell membrane [4], resulting in a significant increase of necrosis and cell debris after cryopreservation. In group B1, cell survival increased significantly, indicating that DMSO at a concentration of 10% (1× ionic concentration with 10% glycerol and 1× ionic concentration with 10% DMSO) has a good protective effect. With an increase of the concentration of DMSO and sodium chloride (group B2, 50%), cell viability decreased significantly with evident apoptosis, but without an increase in necrosis and cell debris, suggesting that a high concentration of cyroprotectant might cause cell damage leading to apoptosis. Thus, it was appropriate to cryopreserve rat hind limbs with 10% DMSO in this study.

The changes to biological tissues at freezing temperatures are complex, and the appropriate selection and application of a cryoprotective substance is critical to cryopreservation [2,3,24]. Many different theories have been proposed for the cause of lethal cell damage during the process of cryopreservation. Mazur *et al*. [25] proposed the two-factor theory, and conjectured that there were two independent factors causing cryo-damage: intracellular cryo-damage caused by intracellular ice formation and solution damage caused by a slow cooling rate. As the temperature decreases there is a loss of intracellular water and increase in the concentration of electrolytes, and in simplistic terms, as biological tissues freeze, ice crystal formation and protein denaturation irreparably damage the cellular structure [2,3,24]. While the exact mechanisms have yet to be fully elucidated, cryoprotective substances prevent ice crystal formation and protein denaturation during cryopreservation. In part, the formation of ice crystals is prevented by an increase in the viscosity of intra- and extracellular fluid [2,3,24]. In general, cryoprotectants can be classified into diffusible substances that cross the cell membrane such as DMSO, glycerol, and 1, 2-propanediol, and non-diffusable substances that to not cross the cell membrane such as hydroxyethyl starch and polyvinylpyroldone [2]. Different types of tissue require different agents for successful cryopreservation, and the dilution of the cryopreservative and length of time the tissue is in contact with the cryopreservative prior to reaching a freezing temperature both affect the integrity of the tissue during preservation [2,3,24].

Cooling rate is another important variable that affects the success of tissue cryopreservation. If the cooling rate is too fast, intracellular ice is more likely to form, and if the cooling rate is too slow cellular injury from the cryoperservative solution can occur [3,4,25]. The optimum cooling rate can vary greatly based on the type of cells, and depends on factors such as whether the process of water penetration can keep up with the cooling speed, the ratio between the surface area and the volume of the cell, and the permeability of the cell membrane [3]. In general, small cells with simpler structure will have a higher optimal cooling rate, approaching 10^3^°C/minute or higher; whereas the optimal cooling rate for large cells with complex structures might be 0.1 to 9°C/minute [3,26]. Thus, for successful cryopreservation, there is a best match of cryopreservative and cooling rate for each type of tissue. This is one of the reasons that skeleton muscle is difficult to preserve via cryopreservation as compared to non-muscle tissues such as skin and bone tissue. In this study, the survival rate of the transfected osteocytes was 88.8 ± 0.9% after cryopreservation.

Modern cryobiological theory holds that the most dangerous temperature zone during cryopreservation is 0 to -60°C, especially 0 to -10°C, because intercellular ice accumulates at these temperatures and cell damage occurs within this temperature zone during the process of cooling or thawing [3,19,27]. Moreover, it has been reported that thawing too slowly induces recrystallization of the intercellular ice, which results in fatal injury to the cell [28]. Therefore, the rate of temperature decrease, especially from room temperature to -60°C, should be controlled by the use of a cooling device. In this study, a slow cooling curve was designed between 4°C and -10°C, the temperature was balanced for 15 minutes to complete the formation of the intercellular ice, and then the temperature was decreased quickly to -40°C. During this process, reconstruction of the intracellular ice is still in progress. After the reconstruction was finished, the temperature was reduced at a faster rate to -140°C, and then the limb was placed in liquid nitrogen.

In the current study, replantation failed in all six rats in the above-knee amputation group. The reason is that the amputated limb became extremely swollen after blood supply was regained, which was especially obvious two hours after reperfusion. At this time, the blood vessels were compressed and blood supply to the limb could not be restored. The failure was mainly due to the impact of skeletal muscle on replantation. The muscle was damaged during the process of deep hypothermia preservation, rewarming, and recirculation, and became swollen as a result of injury during the cryopreservation and rewarming process and ischemia-reperfusion injury after blood flow was restored. Fasciotomy would not likely have prevented the compromised blood flow as the replanted muscles were only loosely sutured. In our prior study using a rat model of limb cryopreservation, transmission electron microscopic observation of muscle that had been cryopreserved and thawed showed that mitochondrial swelling was present within the muscle fibers, most muscle fibers were disorganized, glycogen granules were reduced, the number of mitochondrial cristae were decreased, the myofilaments became loose, the z-line became obscured or disappeared, and autolysis of myeloid bodies occurred [19].

There are a number of limitations of this study. First, the number of animals used was small. Western blotting and RT-PCR to look for stress proteins at the posttranslational level and protein interactions were not performed, and molecular markers of apoptosis were not examined. Lastly, there was no control group studied.

## Conclusion

In conclusion, the results of this study showed that limbs with a minimal amount of muscle tissue can be successfully cryopreserved and replanted. Because of its complexity, cryopreservation of muscle tissue is challenging and injury during the cryopreservation process and from reperfusion results in swelling and elimination of blood flow. The successful replantation of amputated limbs in the rats performed in the current study may provide useful information for future study of cryopreservation of human organs and composite tissues.

## Abbreviations

DMSO: dimethyl sulfoxide; FBS: fetal bovine serum.

## Competing interests

All authors have no potential competing interests.

## Authors’ contributions

ZW participated in research design and the performance of the research; BH participated in research design, the writing of the paper, the performance of the research and data analysis; YD, YS, LZ and XZ participated in the performance of the research; ZZ participated in the writing of the paper and data analysis. All authors approved the final version of manuscript.
